# A Dictionary of Practical Medicine, Comprising General Pathology, the Nature and Treatment of Diseases, Morbid Structures, &c.

**Published:** 1844-11

**Authors:** 


*A Dictionary of Practical Medicine, comprising General Pathology, the Nature and Treatment of Diseases, Morbid Structures, &c.* By JAMES COPLAND, M. D., F. R. S. Edited, with additions, by CHARLES A. LEE, M. D.—New York, Henry G. Langley, 1844. (From the Publishers.)

This valuable work is to be published in monthly parts, the whole to be comprised in 20 numbers; price $10. The additions by the American editor, will supply to the work references to the works of American authors, and also, articles upon diseases peculiarly incident to our own country. The editor promises to leave the original text untouched. This we are glad to see, for it is not always that *corrections*, so called, are *improvements.* In the part before us, (Part 1.) the articles upon Abscess and Abortion are well worth the price of the whole number.—Ed.

